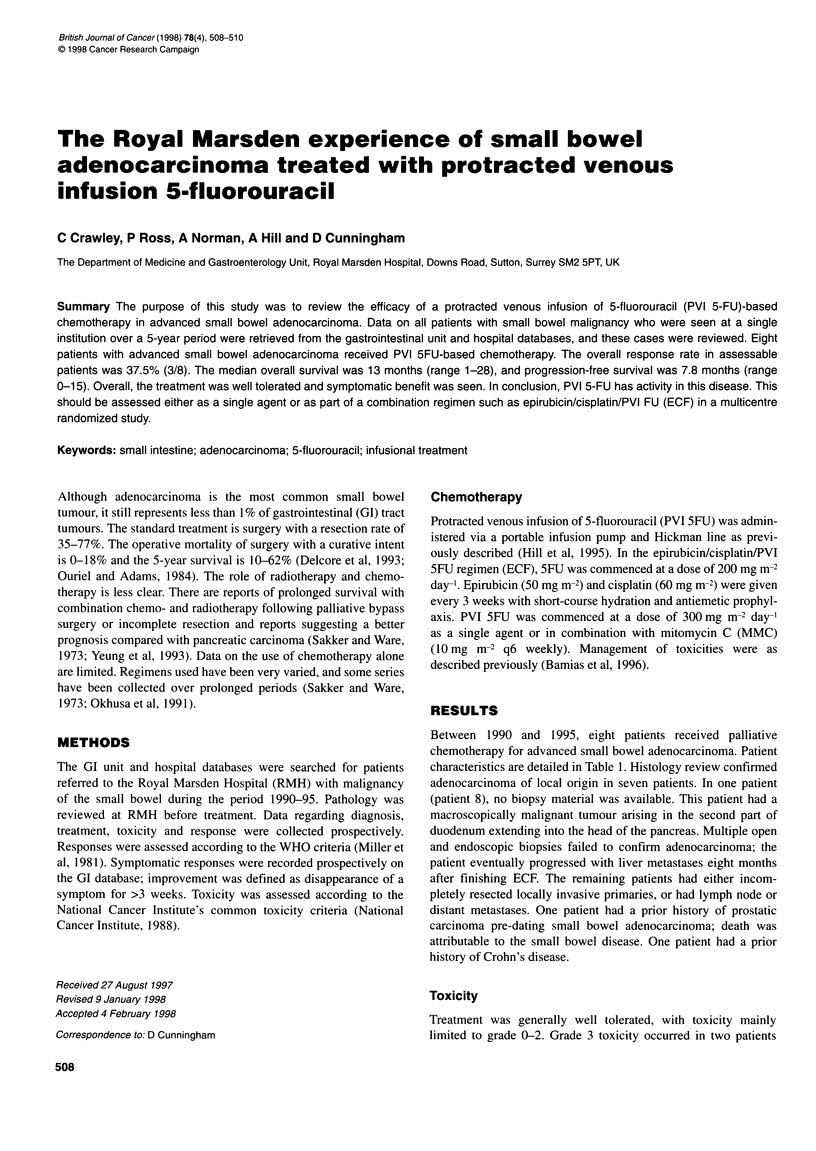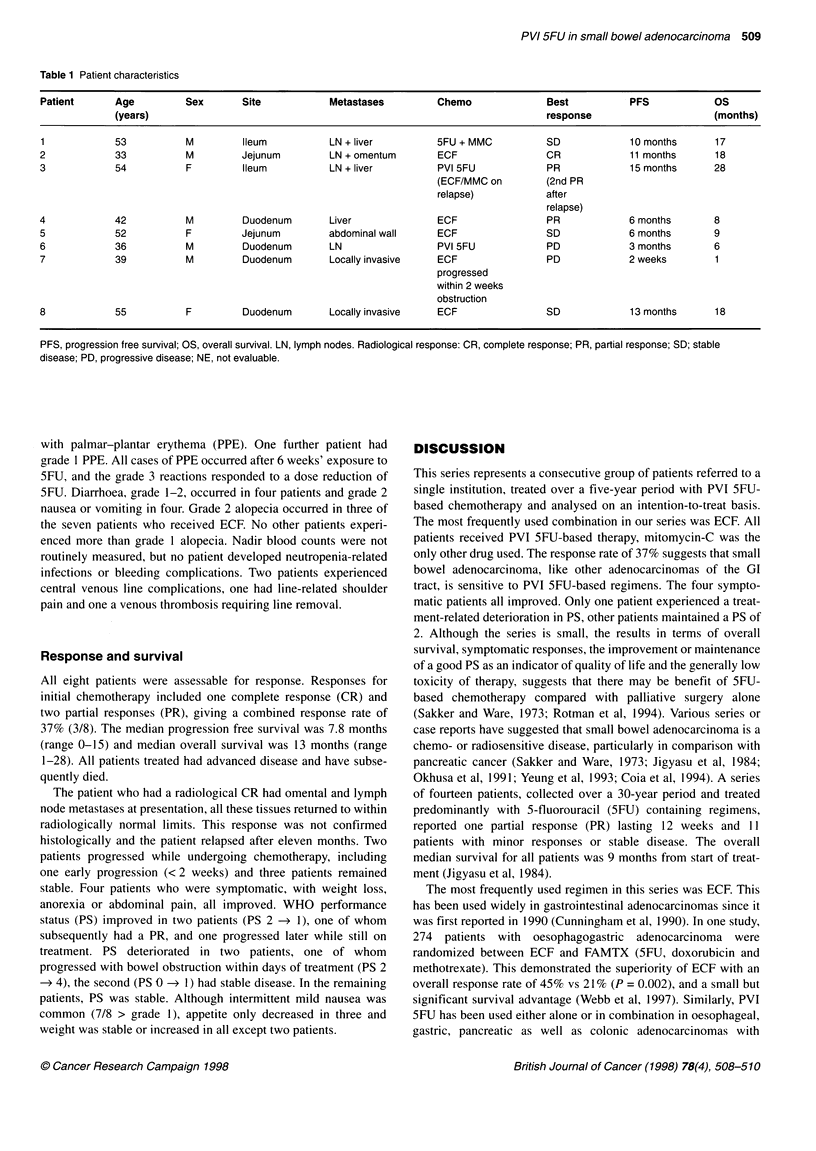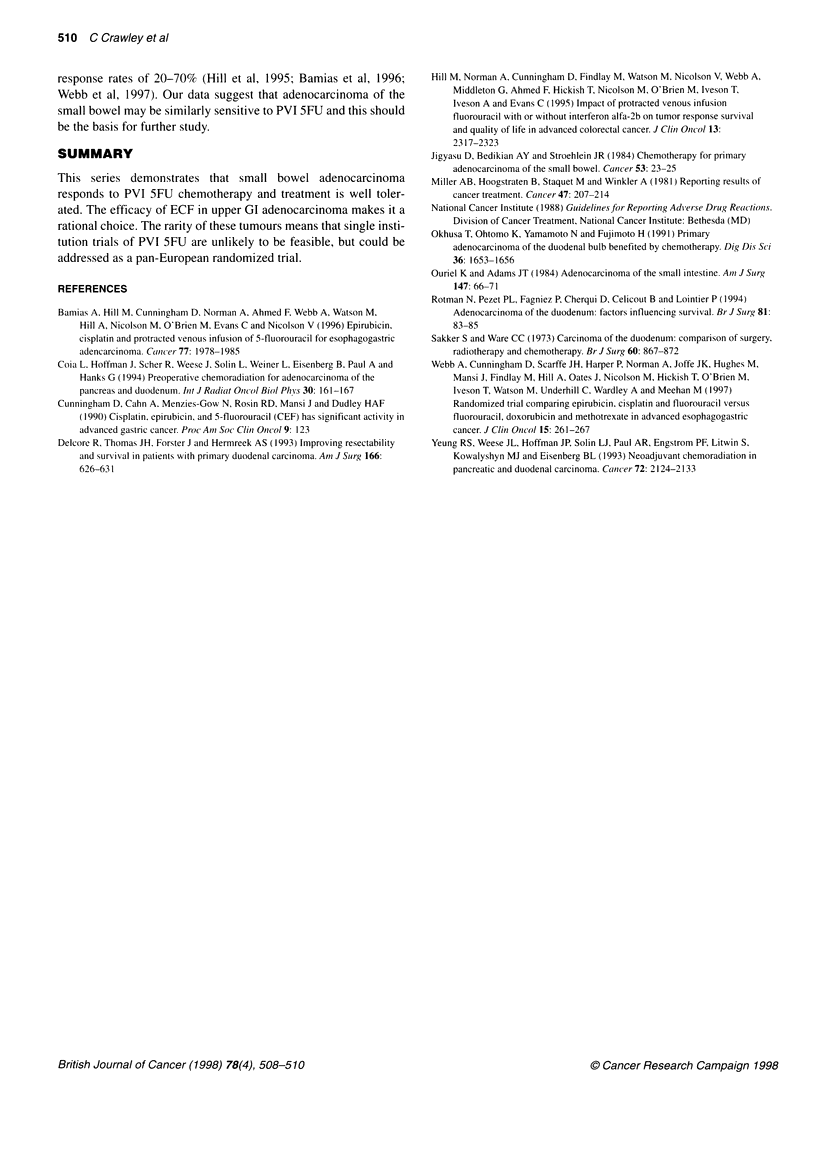# The Royal Marsden experience of a small bowel adenocarcinoma treated with protracted venous infusion 5-fluorouracil.

**DOI:** 10.1038/bjc.1998.523

**Published:** 1998-08

**Authors:** C. Crawley, P. Ross, A. Norman, A. Hill, D. Cunningham

**Affiliations:** The Department of Medicine, Royal Marsden Hospital, Sutton, Surrey, UK.

## Abstract

The purpose of this study was to review the efficacy of a protracted venous infusion of 5-fluorouracil (PVI 5-FU)-based chemotherapy in advanced small bowel adenocarcinoma. Data on all patients with small bowel malignancy who were seen at a single institution over a 5-year period were retrieved from the gastrointestinal unit and hospital databases, and these cases were reviewed. Eight patients with advanced small bowel adenocarcinoma received PVI 5FU-based chemotherapy. The overall response rate in assessable patients was 37.5% (3/8). The median overall survival was 13 months (range 1-28), and progression-free survival was 7.8 months (range 0-15). Overall, the treatment was well tolerated and symptomatic benefit was seen. In conclusion, PVI 5-FU has activity in this disease. This should be assessed either as a single agent or as part of a combination regimen such as epirubicin/cisplatin/PVI FU (ECF) in a multicentre randomized study.


British Journal of Cancer (1998) 78(4), 508–510
© 1998 Cancer Research Campaign

### The Royal Marsden experience of small bowel adenocarcinoma treated with protracted venous infusion 5-fluorouracil

**C Crawley, P Ross, A Norman, A Hill and D Cunningham**

The Department of Medicine and Gastroenterology Unit, Royal Marsden Hospital, Downs Road, Sutton, Surrey SM2 5PT, UK

**Summary** The purpose of this study was to review the efficacy of a protracted venous infusion of 5-fluorouracil (PVI 5-FU)-based chemotherapy in advanced small bowel adenocarcinoma. Data on all patients with small bowel malignancy who were seen at a single institution over a 5-year period were retrieved from the gastrointestinal unit and hospital databases, and these cases were reviewed. Eight patients with advanced small bowel adenocarcinoma received PVI 5FU-based chemotherapy. The overall response rate in assessable patients was 37.5% (3/8). The median overall survival was 13 months (range 1–28), and progression-free survival was 7.8 months (range 0–15). Overall, the treatment was well tolerated and symptomatic benefit was seen. In conclusion, PVI 5-FU has activity in this disease. This should be assessed either as a single agent or as part of a combination regimen such as epirubicin/cisplatin/PVI FU (ECF) in a multicentre randomized study.

**Keywords:** small intestine; adenocarcinoma; 5-fluorouracil; infusional treatment

Although adenocarcinoma is the most common small bowel tumour, it still represents less than 1% of gastrointestinal (GI) tract tumours. The standard treatment is surgery with a resection rate of 35–77%. The operative mortality of surgery with a curative intent is 0–18% and the 5-year survival is 10–62% (Delcore et al, 1993; Ouriel and Adams, 1984). The role of radiotherapy and chemotherapy is less clear. There are reports of prolonged survival with combination chemo- and radiotherapy following palliative bypass surgery or incomplete resection and reports suggesting a better prognosis compared with pancreatic carcinoma (Sakker and Ware, 1973; Yeung et al, 1993). Data on the use of chemotherapy alone are limited. Regimens used have been very varied, and some series have been collected over prolonged periods (Sakker and Ware, 1973; Okhusa et al, 1991).

### METHODS

The GI unit and hospital databases were searched for patients referred to the Royal Marsden Hospital (RMH) with malignancy of the small bowel during the period 1990–95. Pathology was reviewed at RMH before treatment. Data regarding diagnosis, treatment, toxicity and response were collected prospectively. Responses were assessed according to the WHO criteria (Miller et al, 1981). Symptomatic responses were recorded prospectively on the GI database; improvement was defined as disappearance of a symptom for >3 weeks. Toxicity was assessed according to the National Cancer Institute's common toxicity criteria (National Cancer Institute, 1988).

*Received 27 August 1997*
*Revised 9 January 1998*
*Accepted 4 February 1998*

*Correspondence to:* D Cunningham

### Chemotherapy

Protracted venous infusion of 5-fluorouracil (PVI 5FU) was administered via a portable infusion pump and Hickman line as previously described (Hill et al, 1995). In the epirubicin/cisplatin/PVI 5FU regimen (ECF), 5FU was commenced at a dose of 200 mg $m^{-2}$ $day^{-1}$. Epirubicin (50 mg $m^{-2}$) and cisplatin (60 mg $m^{-2}$) were given every 3 weeks with short-course hydration and antiemetic prophylaxis. PVI 5FU was commenced at a dose of 300 mg $m^{-2}$ $day^{-1}$ as a single agent or in combination with mitomycin C (MMC) (10 mg $m^{-2}$ q6 weekly). Management of toxicities were as described previously (Bamias et al, 1996).

### RESULTS

Between 1990 and 1995, eight patients received palliative chemotherapy for advanced small bowel adenocarcinoma. Patient characteristics are detailed in Table 1. Histology review confirmed adenocarcinoma of local origin in seven patients. In one patient (patient 8), no biopsy material was available. This patient had a macroscopically malignant tumour arising in the second part of duodenum extending into the head of the pancreas. Multiple open and endoscopic biopsies failed to confirm adenocarcinoma; the patient eventually progressed with liver metastases eight months after finishing ECF. The remaining patients had either incompletely resected locally invasive primaries, or had lymph node or distant metastases. One patient had a prior history of prostatic carcinoma pre-dating small bowel adenocarcinoma; death was attributable to the small bowel disease. One patient had a prior history of Crohn's disease.

### Toxicity

Treatment was generally well tolerated, with toxicity mainly limited to grade 0–2. Grade 3 toxicity occurred in two patients

**Table 1** Patient characteristics

| Patient | Age (years) | Sex | Site | Metastases | Chemo | Best response | PFS | OS (months) |
|---|---|---|---|---|---|---|---|---|
| 1 | 53 | M | Ileum | LN + liver | 5FU + MMC | SD | 10 months | 17 |
| 2 | 33 | M | Jejunum | LN + omentum | ECF | CR | 11 months | 18 |
| 3 | 54 | F | Ileum | LN + liver | PVI 5FU (ECF/MMC on relapse) | PR (2nd PR after relapse) | 15 months | 28 |
| 4 | 42 | M | Duodenum | Liver | ECF | PR | 6 months | 8 |
| 5 | 52 | F | Jejunum | abdominal wall | ECF | SD | 6 months | 9 |
| 6 | 36 | M | Duodenum | LN | PVI 5FU | PD | 3 months | 6 |
| 7 | 39 | M | Duodenum | Locally invasive | ECF progressed within 2 weeks obstruction | PD | 2 weeks | 1 |
| 8 | 55 | F | Duodenum | Locally invasive | ECF | SD | 13 months | 18 |

PFS, progression free survival; OS, overall survival. LN, lymph nodes. Radiological response: CR, complete response; PR, partial response; SD; stable disease; PD, progressive disease; NE, not evaluable.

with palmar–plantar erythema (PPE). One further patient had grade 1 PPE. All cases of PPE occurred after 6 weeks' exposure to 5FU, and the grade 3 reactions responded to a dose reduction of 5FU. Diarrhoea, grade 1–2, occurred in four patients and grade 2 nausea or vomiting in four. Grade 2 alopecia occurred in three of the seven patients who received ECF. No other patients experienced more than grade 1 alopecia. Nadir blood counts were not routinely measured, but no patient developed neutropenia-related infections or bleeding complications. Two patients experienced central venous line complications, one had line-related shoulder pain and one a venous thrombosis requiring line removal.

### Response and survival

All eight patients were assessable for response. Responses for initial chemotherapy included one complete response (CR) and two partial responses (PR), giving a combined response rate of 37% (3/8). The median progression free survival was 7.8 months (range 0–15) and median overall survival was 13 months (range 1–28). All patients treated had advanced disease and have subsequently died.

The patient who had a radiological CR had omental and lymph node metastases at presentation, all these tissues returned to within radiologically normal limits. This response was not confirmed histologically and the patient relapsed after eleven months. Two patients progressed while undergoing chemotherapy, including one early progression (< 2 weeks) and three patients remained stable. Four patients who were symptomatic, with weight loss, anorexia or abdominal pain, all improved. WHO performance status (PS) improved in two patients (PS 2 → 1), one of whom subsequently had a PR, and one progressed later while still on treatment. PS deteriorated in two patients, one of whom progressed with bowel obstruction within days of treatment (PS 2 → 4), the second (PS 0 → 1) had stable disease. In the remaining patients, PS was stable. Although intermittent mild nausea was common (7/8 > grade 1), appetite only decreased in three and weight was stable or increased in all except two patients.

### DISCUSSION

This series represents a consecutive group of patients referred to a single institution, treated over a five-year period with PVI 5FU-based chemotherapy and analysed on an intention-to-treat basis. The most frequently used combination in our series was ECF. All patients received PVI 5FU-based therapy, mitomycin-C was the only other drug used. The response rate of 37% suggests that small bowel adenocarcinoma, like other adenocarcinomas of the GI tract, is sensitive to PVI 5FU-based regimens. The four symptomatic patients all improved. Only one patient experienced a treatment-related deterioration in PS, other patients maintained a PS of 2. Although the series is small, the results in terms of overall survival, symptomatic responses, the improvement or maintenance of a good PS as an indicator of quality of life and the generally low toxicity of therapy, suggests that there may be benefit of 5FU-based chemotherapy compared with palliative surgery alone (Sakker and Ware, 1973; Rotman et al, 1994). Various series or case reports have suggested that small bowel adenocarcinoma is a chemo- or radiosensitive disease, particularly in comparison with pancreatic cancer (Sakker and Ware, 1973; Jigyasu et al, 1984; Okhusa et al, 1991; Yeung et al, 1993; Coia et al, 1994). A series of fourteen patients, collected over a 30-year period and treated predominantly with 5-fluorouracil (5FU) containing regimens, reported one partial response (PR) lasting 12 weeks and 11 patients with minor responses or stable disease. The overall median survival for all patients was 9 months from start of treatment (Jigyasu et al, 1984).

The most frequently used regimen in this series was ECF. This has been used widely in gastrointestinal adenocarcinomas since it was first reported in 1990 (Cunningham et al, 1990). In one study, 274 patients with oesophagogastric adenocarcinoma were randomized between ECF and FAMTX (5FU, doxorubicin and methotrexate). This demonstrated the superiority of ECF with an overall response rate of 45% vs 21% ($P = 0.002$), and a small but significant survival advantage (Webb et al, 1997). Similarly, PVI 5FU has been used either alone or in combination in oesophageal, gastric, pancreatic as well as colonic adenocarcinomas with

© Cancer Research Campaign 1998

response rates of 20–70% (Hill et al, 1995; Bamias et al, 1996; Webb et al, 1997). Our data suggest that adenocarcinoma of the small bowel may be similarly sensitive to PVI 5FU and this should be the basis for further study.

### SUMMARY

This series demonstrates that small bowel adenocarcinoma responds to PVI 5FU chemotherapy and treatment is well tolerated. The efficacy of ECF in upper GI adenocarcinoma makes it a rational choice. The rarity of these tumours means that single institution trials of PVI 5FU are unlikely to be feasible, but could be addressed as a pan-European randomized trial.

### REFERENCES

Bamias A, Hill M, Cunningham D, Norman A, Ahmed F, Webb A, Watson M, Hill A, Nicolson M, O'Brien M, Evans C and Nicolson V (1996) Epirubicin, cisplatin and protracted venous infusion of 5-fluorouracil for esophagogastric adencarcinoma. *Cancer* **77**: 1978–1985

Coia L, Hoffman J, Scher R, Weese J, Solin L, Weiner L, Eisenberg B, Paul A and Hanks G (1994) Preoperative chemoradiation for adenocarcinoma of the pancreas and duodenum. *Int J Radiat Oncol Biol Phys* **30**: 161–167

Cunningham D, Cahn A, Menzies-Gow N, Rosin RD, Mansi J and Dudley HAF (1990) Cisplatin, epirubicin, and 5-fluorouracil (CEF) has significant activity in advanced gastric cancer. *Proc Am Soc Clin Oncol* **9**: 123

Delcore R, Thomas JH, Forster J and Hermreek AS (1993) Improving resectability and survival in patients with primary duodenal carcinoma. *Am J Surg* **166**: 626–631

Hill M, Norman A, Cunningham D, Findlay M, Watson M, Nicolson V, Webb A, Middleton G, Ahmed F, Hickish T, Nicolson M, O'Brien M, Iveson T, Iveson A and Evans C (1995) Impact of protracted venous infusion fluorouracil with or without interferon alfa-2b on tumor response survival and quality of life in advanced colorectal cancer. *J Clin Oncol* **13**: 2317–2323

Jigyasu D, Bedikian AY and Stroehlein JR (1984) Chemotherapy for primary adenocarcinoma of the small bowel. *Cancer* **53**: 23–25

Miller AB, Hoogstraten B, Staquet M and Winkler A (1981) Reporting results of cancer treatment. *Cancer* **47**: 207–214

National Cancer Institute (1988) *Guidelines for Reporting Adverse Drug Reactions*. Division of Cancer Treatment, National Cancer Institute: Bethesda (MD)

Okhusa T, Ohtomo K, Yamamoto N and Fujimoto H (1991) Primary adenocarcinoma of the duodenal bulb benefited by chemotherapy. *Dig Dis Sci* **36**: 1653–1656

Ouriel K and Adams JT (1984) Adenocarcinoma of the small intestine. *Am J Surg* **147**: 66–71

Rotman N, Pezet PL, Fagniez P, Cherqui D, Celicout B and Lointier P (1994) Adenocarcinoma of the duodenum: factors influencing survival. *Br J Surg* **81**: 83–85

Sakker S and Ware CC (1973) Carcinoma of the duodenum: comparison of surgery, radiotherapy and chemotherapy. *Br J Surg* **60**: 867–872

Webb A, Cunningham D, Scarffe JH, Harper P, Norman A, Joffe JK, Hughes M, Mansi J, Findlay M, Hill A, Oates J, Nicolson M, Hickish T, O'Brien M, Iveson T, Watson M, Underhill C, Wardley A and Meehan M (1997) Randomized trial comparing epirubicin, cisplatin and fluorouracil versus fluorouracil, doxorubicin and methotrexate in advanced esophagogastric cancer. *J Clin Oncol* **15**: 261–267

Yeung RS, Weese JL, Hoffman JP, Solin LJ, Paul AR, Engstrom PF, Litwin S, Kowalyshyn MJ and Eisenberg BL (1993) Neoadjuvant chemoradiation in pancreatic and duodenal carcinoma. *Cancer* **72**: 2124–2133

© Cancer Research Campaign 1998